# In-office needle arthroscopy is a cost-effective alternative for operating room diversion in medial meniscectomy: a financial analysis

**DOI:** 10.1186/s13018-023-03866-7

**Published:** 2023-06-15

**Authors:** Aazad Abbas, Ajay Shah, Johnathan R. Lex, Jihad Abouali, Jay Toor

**Affiliations:** 1 Temerty Faculty of Medicine, University of Toronto, 1 King’s College Circle, Toronto, ON M5S 1A8 Canada; 2 Division of Orthopaedic Surgery, University of Toronto, 149 College Street Room 508-A, Toronto, ON M5T 1P5 Canada; 3 Division of Orthopaedic Surgery, Michael Garron Hospital, 825 Coxwell Avenue, Toronto, ON M4C 3E7 Canada

**Keywords:** Arthroscopy, Cost-effectiveness, Needle, In-office, Wait times, Efficiency

## Abstract

**Background:**

In-office needle arthroscopy (IONA) has been described as a diagnostic alternative to magnetic resonance imaging (MRI) for intra-articular pathology. However, few studies have analyzed its impact on cost and wait times when used as a therapeutic intervention. The purpose of this study was to investigate the impact on cost and wait times associated with offering IONA for partial medial meniscectomy as an alternative to traditional operating room (OR) arthroscopy for patients with irreparable medial meniscus tears on MRI.

**Methods:**

Two models were created comparing the existing care pathway (current state) to a proposed future state utilizing IONA. Data sources were accounting data from an academically affiliated hospital in Canada and supplemented with literature values. A Monte Carlo simulation combined with DuPont analysis running 10,000 simulations was conducted to calculate the revenue, expenses, profits, and effect on surgical waitlists (i.e., throughput) between the states. Sensitivity analyses examined the influence of patient preference and revision rates on profit and throughput. Two-sample Student’s *t* test was performed (*p* < .05).

**Results:**

An average of 198 (standard deviation (SD) 31) patients underwent arthroscopic meniscectomy or repair each year from 2016 to 2020. The IONA revision rate was calculated as 20.3%. Compared to the current state, annual expenses in the IONA pathway were significantly reduced ($266,912.68 versus $281,415.23, *p* < .0001), while improving throughput by 21.2% (3.54%). Sensitivity analysis revealed 10% of patients need to select IONA over traditional OR arthroscopy with the revision rate remaining below 40% for the proposed state profit to be higher than the current state.

**Conclusions:**

IONA is a cost-effective alternative to traditional OR arthroscopy in patients undergoing partial medial meniscectomy. The next steps are to assess patients’ perceptions of IONA as an alternative to traditional OR arthroscopy, and to carry out clinical trials to determine the efficacy, patient-reported outcome metrics, and complications of IONA.

## Introduction

The efficiency of orthopedic care delivery with respect to wait times and systemic costs is extremely concerning. Canadian orthopedic patients experience the longest wait times of any G7 country, yet perioperative surgical care constitutes a significant portion of a hospital’s budget [1–3]. This problem is exacerbated by the rapidly increasing demand for surgery which, combined with a backlog of referrals and surgeries due to the COVID-19 pandemic hiatus, is magnifying these issues [4]. This suggests that more effort should be dedicated to exploring methodology to improve surgical throughput and reducing costs such as the diversion of procedures away from the operating room (OR) into a clinical setting.

The concept of OR diversion to the office setting has been implemented successfully for laryngopharyngeal [5, 6], endovascular [7, 8], cystoscopic [9, 10], and minor hand surgical procedures, such as carpal tunnel release [11, 12]. Diversion from the OR to the clinic has the potential to free up surgical time, which is a highly constrained and expensive resource [11, 13]. In contrast, clinic time is not typically constrained, allowing for considerable throughput which can dramatically lower wait lists. To date, few attempts to divert orthopedic surgery procedures from the OR have been attempted, due to the relatively invasive nature of these surgeries. However, technology is rapidly evolving, and new techniques are being explored with the potential for application to procedures amenable to OR diversion.

In-office needle arthroscopy (IONA) is an emerging technology that has been primarily studied as a diagnostic tool. Recent evidence shows that devices such as the NanoScope (Arthrex Inc., Naples, FL) are a cost-effective alternative to hospital- and community-based MRI with comparable accuracy [14, 15]. It is increasingly popular as both a diagnostic and therapeutic tool in the literature, particularly in patients with contra-indications to MRI or surgical arthroscopy such as those who are pregnant or who cannot tolerate anesthesia [16, 17]. Recent procedure guides detailing IONA medial meniscectomy suggest a potential for OR diversion, primarily in the office setting [18–20].

Knee arthroscopy with meniscectomy is the third most common orthopedic surgery performed after total knee and hip arthroplasty, comprising up to 16.6% of all procedures [21, 22]. In Ontario, 32,000 knee arthroscopies are conducted annually, and 97% are in the ambulatory surgical setting [23]. Knee arthroscopy is currently a quality-based procedure (QBP) in Ontario, a local application of the “Value-Based Care” or “bundled payment” concept [24]. QBPs and similar bundled care models are intended to drive efficiency. Due to targeted government reimbursement per procedure, analogous to a traditional business model’s revenue and hospital case costing of expenses, bundled payment models are highly amenable to comprehensive business evaluations of efficiency such as DuPont analysis [24].

Given the high case volume of knee arthroscopy as well as the potential amenability to be diverted away from the OR to the office setting, IONA has the potential to generate considerable improvements in healthcare system efficiency with respect to throughput and cost savings. To date, no studies have investigated the potential cost savings associated with offering IONA for isolated medial meniscectomy. As such, the purpose of this study is to investigate the cost savings and impact on waiting times on a mid-sized Canadian community hospital if IONA is offered as an alternative to traditional OR arthroscopy for medial meniscal tears.

## Methods

This project was exempt from Institutional Review Ethics Board review under Section 2.5 of the Tri Council Policy Statement. This study was carried out in a mid-sized Canadian community hospital. De-identified data were obtained from the OR booking schedule and the hospital’s case costing data and were supplemented with data from the literature. This information was used to generate two financial operational models comparing the existing care pathway of medial meniscus tears, i.e., the current state to a proposed future state utilizing IONA as an option for OR diversion for medial meniscus tears.

### Process mapping

To develop a comprehensive understanding and accurate representation of the quantifiable operations involved in the current state for medial meniscus tear care, process mapping was performed that describes the journey of a patient from when they present with knee pain to their general practitioner until case resolution. This technique was then repeated to create a second process map describing the hypothetical proposed state whereby OR diversion may be conducted utilizing IONA.

### DuPont financial modeling

Once the respective process maps for each state were determined, each process map was translated into a DuPont decision tree. DuPont analysis disaggregates profit into revenue and expenses with their respective contributing operational components [25]. Decision tree analysis was used to determine the number of patients in each state determined by the process mapping. The results of each model were used to compare the current state to the proposed state according to profit and profit margin using standard generally accepted accounting principles (GAAP) [26]. All analysis was conducted using Microsoft Excel software (Redmond, WA).

#### Data collection

To accurately determine the total number of patients which would be eligible for this care pathway at our institution, the OR booking scheduling for arthroscopy and meniscectomy/repair over a 4-year period (2016–2020) was reviewed. To quantify the billing and costs associated with each node in the decision tree, the hospital’s case costing data were also reviewed over the same period. To ensure the generalizability of this study, the pooled percentage of patients that would be distributed within each arm of the decision tree for each state was determined from a literature review.

There are currently no published clinical studies that report on the safety or efficacy of IONA for medial meniscectomy. Therefore, some assumptions from the literature were made. For the current state, the percentages of MRIs indicating repairable or irreparable as well as the percentages of meniscectomies, repairs, and diagnostic arthroscopies for each arm of the decision tree were determined from a literature search. This assumption was made to ensure the decision tree for the current state is generalizable. For the proposed state, the number of patients requiring an additional full OR after an IONA procedure—defined as IONA revision—was also determined from a literature search. The IONA revision rate was assumed to be the same as the traditional arthroscopy rate where the MRI suggests irreparable but is repaired since it would be the same procedure as an arthroscopy with meniscal repair. This assumption was made as the rate for IONA revisions is currently unknown. The search terms included “repairability,” “medial meniscus tear,” and “MRI.” Primary studies reporting on the accuracy for predicted resection or repairability based on preoperative MRI scans of isolated meniscus tears and eventual treatment with repair or resection were included. In addition, false positive rates of preoperative MRI scans indicated isolated meniscus tears were included to determine diagnostic arthroscopy percentages. Results were pooled to estimate a pooled sensitivity, specificity, and percentage for each arm of the decision tree in both the current and proposed states.

#### Financial modeling for each state

For the current state, QBP compensation per procedure for meniscectomy, repair, and diagnostic arthroscopy repair was summed to determine revenue. Expenses were determined from case costing data for all three procedures, respectively. Costs were all inclusive and included equipment costs, saline usage, sterilization, and anesthesia costs.

For the proposed state, the current state model was adjusted to include the additional operational costs associated with IONA. These include (1) disposable costs per IONA and (2) cost per OR for patients requiring an IONA revision. All currency was reported in Canadian dollars (CAD).

#### Model heuristics

**Fig. 1:**
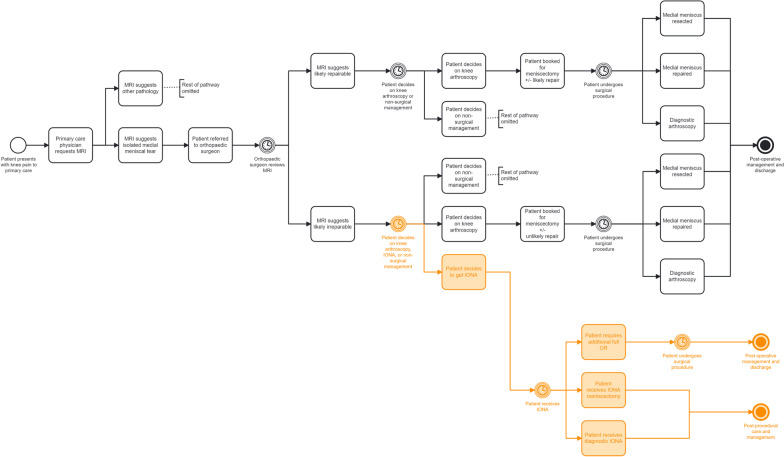
Current (black) versus proposed (black and orange) pathway for patients with a medial meniscus tear on MRI

For simplicity of modeling, the following heuristics were assumed:

1. All patients in this model do not opt for non-surgical intervention (Fig. 1). This is accurate based on our institution as only the patients who were booked for OR meniscectomy or repair were included in the model.
2. All patients who would require an extra OR after IONA (i.e., revision IONA) would require a repair, since an IONA meniscectomy or diagnostic IONA was not possible. This is consistent with current practice using traditional arthroscopy.
3. All patients who required a repair after receiving IONA were repaired, i.e., they could not decide to have a meniscectomy instead of repair. This is to ensure there was no crossover between different care pathways.
4. Patients who qualify for the IONA care pathway are the same who would qualify for traditional arthroscopy based on demographic factors, symptoms, amount of arthritis, and tear type. This was to ensure a direct comparison with arthroscopy and is consistent with current IONA recommendations and arthroscopy practice [14–20].
5. QBP billing for IONA meniscectomy, diagnostic, or revision would be equivalent to the current QBP billing for OR meniscectomy. This is to ensure a one-to-one comparison with current billing standards.
6. When presented with the option to choose between OR meniscectomy ± unlikely repair or IONA, 50% of patients would select each. This was done as no literature value has been established for this node. Sensitivity analysis was done to further explore this effect.

#### Stochastic modeling

For the model to be generalizable, the number of patients undergoing each of the six possible procedures (OR meniscectomy, OR meniscus repair, OR diagnostic arthroscopy, IONA meniscectomy, IONA revision, diagnostic IONA) was calculated by incorporating a Monte Carlo simulation (MCS). *N* = 10,000 simulations were conducted using the MCS on these variables based on the mean and standard deviation of the total number of patients seen at our institution over the years 2016–2020. The mean and standard deviation of the MCS for the revenue, expenses, profit, and profit margin for each state were determined (MCS-DuPont financial model). The paired two-sample Student’s *t* test was used to determine the statistical significance between the revenue, expenses, and profit between the current and proposed states. Statistical significance was set at *p* < 0.05.

### Waitlist comparison

A potential decrease in the surgical waitlist may be approximated by the number of patients that may be diverted away from the OR (throughput). This throughput may be modeled by the percentage of patients that may be given a successful IONA, which is defined by the following equation:

1 $${\text{Throughput}} = N_{{{\text{IONA}}}} /N,$$

where $N_{{{\text{IONA}}}}$ is the number of patients that may be successfully treated with IONA and $N$ is the total number of patients. $N_{{{\text{IONA}}}}$ may be further broken down by the following equation:

2 $$N_{{{\text{IONA}}}} = \mathop \sum \limits_{n\varepsilon N}^{N} n_{{{\text{CSO}}}} - \mathop \sum \limits_{n\varepsilon N}^{N} n_{{{\text{PSO}}}} = N_{{{\text{CSM}}}} + N_{{{\text{CSR}}}} + N_{{{\text{CSDA}}}} - N_{{{\text{PSM}}}} - N_{{{\text{PSR}}}} - N_{{{\text{PSDA}}}} - N_{{{\text{PSRI}}}} ,$$

where $n$ is each patient, $N$ is the total number of patients, $n_{{{\text{CSO}}}}$ is each patient taken to the OR in the current state, $n_{{{\text{PSO}}}}$ is each patient taken to the OR in the proposed state, $N_{{{\text{CSM}}}}$ is the total number of patients who undergo meniscectomy in the current state, $N_{{{\text{CSR}}}}$ is the number of patients who undergo repair in the current state, $N_{{{\text{CSDA}}}}$ is the number of patients who undergo diagnostic arthroscopy in the current state, $N_{{{\text{PSM}}}}$ is the number of patients who undergo meniscectomy in the proposed state, $N_{{{\text{PSR}}}}$ is the number of patients who undergo repair in the proposed state, $N_{{{\text{PSDA}}}}$ is the number of patients who undergo diagnostic arthroscopy in the proposed state, and $N_{{{\text{PSIR}}}}$ is the number of patients who undergo IONA revision in the proposed state. Since IONA can only divert patients whose MRI indicates irreparable, Eq. 2 simplifies to only the patients who are in the irreparable arm of the decision tree. Like the DuPont financial model, the throughput was made generalizable using an MCS with *N* = 10,000 simulations comparing the current and proposed states (MCS-throughput model).

### Sensitivity analysis

A sensitivity analysis was performed to examine the effect of the number of patients who select IONA over meniscectomy and the number of revision meniscectomies after IONA on (1) the profit and profit margin determined by the MCS-DuPont financial model and (2) the throughput (percentage and number) determined by the MCS-throughput model.

## Results

### Process mapping and modeling

**Fig. 2:**
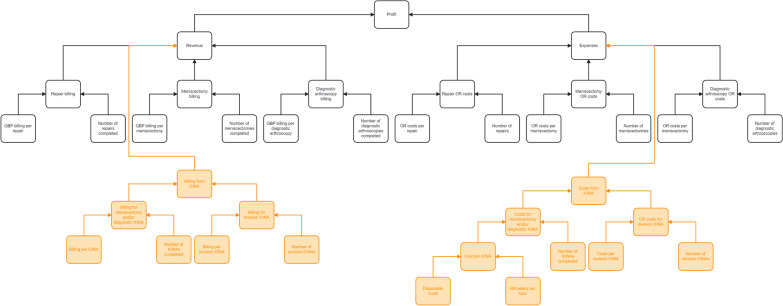
Current (black) and proposed (black and orange) state DuPont financial tree derived from process mapping

A process map of the current state for a patient presenting with knee pain to their general practitioner is described in the black portion of Fig. 1, with the proposed state process map including both the black and orange portions of the figure. The DuPont trees summarizing the variables involved in the current state and proposed state are displayed in Fig. 2, with the black portion representing the current state and the black and orange portion representing the proposed state.

Based on historic data at our institution, an average of 198 patients (SD 31) underwent either a meniscectomy or repair each year between 2016 and 2020. This was used to determine the total number of patients that would be eligible for the care pathways of the current and proposed states.

The prevalence of MRI-confirmed isolated medial meniscal tears among patients with new-onset (< 48 months) symptomatic knee pain without prior abnormality on baseline MRI was reported at 20.0%. A literature review resulted in six studies with 508 patients for the prediction of repairability and resection of meniscus tears from preoperative MRIs, with results summarized in Table 1 [27–32]. Pooled sensitivity and specificity data for the reliability of meniscus resection versus repair suggest that 20.3% of patients undergoing IONA for expected meniscus resection would potentially require a revision of traditional surgical arthroscopy for meniscus repair [32–35].

**Table 1:** Pooled data for probabilistic sequences for each arm of the DuPont decision tree was determined from a literature review [26, 38–42]

Stage	Pooled number of patients (*N*/total per stage)	Pooled percentage of patients (%)	Percentage in each treatment arm (%)^b^
MRI indicates likely repairable	240/508	47.2	
Repaired	173/240	72.1	63.4
Resected	67/240	27.9	24.6
Diagnostic arthroscopy^a^	NA	NA	12.0
MRI indicates likely irreparable	268/508	52.8	
Repaired	62/268	23.1	20.3
Resected	206/268	76.9	67.7
Diagnostic arthroscopy^a^	NA	NA	12.0
False positive^a^	NA	12.0	12.0

^a^Diagnostic arthroscopy percentages are determined from false positives rates on MRI

^b^Treatment arms within each MRI result, i.e., for those patients whose MRI indicates the meniscus is likely repairable, 63.4% of them will be repaired

### Financial and throughput comparison

Financial metrics between the current and proposed states are displayed in Table 2. Revenue for both states was similar (*p* = 0.22), with the current state revenue being $ 248,555.99 (standard deviation $ 39,005.43) and proposed state revenue of $ 249,223.86 (SD $ 39,188.73).

**Table 2:** Comparing financial metrics between the current state and proposed state using heuristics found in the Model Heuristics section

Metric	Current state		Proposed state		Percentage difference (%)	*p* value
	Mean	SD	Mean	SD		
Revenue total	$ 248,555.99	$ 39,005.43	$ 249,223.86	$ 39,188.73	0.3	.22
OR meniscectomy/DA billing	$ 147,440.21	$ 23,160.57	$ 95,438.42	$ 15,037.25	− 35.3	< .0001
OR repair billing	$ 101,115.79	$ 15,857.05	$ 87,993.48	$ 13,824.07	− 13.0	< .0001
IONA meniscectomy/DA billing	NA		$ 53,068.64		NA	
IONA revision billing	NA		$ 12,733.33		NA	
Expenses total	$ 281,415.23	$ 44,157.80	$ 266,912.68	$ 42,093.19	− 5.2	< .0001
OR meniscectomy/DA costs	$ 152,605.31	$ 23,971.92	$ 98,781.80	$ 15,564.04	− 35.3	< .0001
OR repair costs	$ 128,809.92	$ 20,200.06	$ 112,093.60	$ 17,610.29	− 13.0	< .0001
IONA meniscectomy/DA costs	NA		$ 32,111.60	$ 4995.99	NA	
IONA revision costs	NA		$ 23,925.68	$ 4001.85	NA	
Profit	$ (32,859.24)	$ 5153.49	$ (17,678.82)	$ 2921.28	46.2	< .0001
Profit margin	− 13%		− 7%		+ 6%	

However, the reduction in expenses was significant (*p* < 0.0001) at 5.15%, with expenses in the current state being $ 281,415.23 (SD $ 44,157.80) and proposed state of $ 266,912.68 (SD $ 42,093.19), representing $14,502.95 in savings. Accordingly, profit improvement was also significant (*p* < 0.0001) at 46.2%, with current state profit being $ (32,859.24) (SD $ 5,153.49) and proposed state being $ (17,678.82) (SD $ 2,921.28). The addition of IONA into the care pathway of the proposed state produced an average improvement in throughput of 42 patients (SD 7), representing a 21.2% reduction in the number of patients that require an OR procedure.

### Sensitivity analysis

**Fig. 3:**
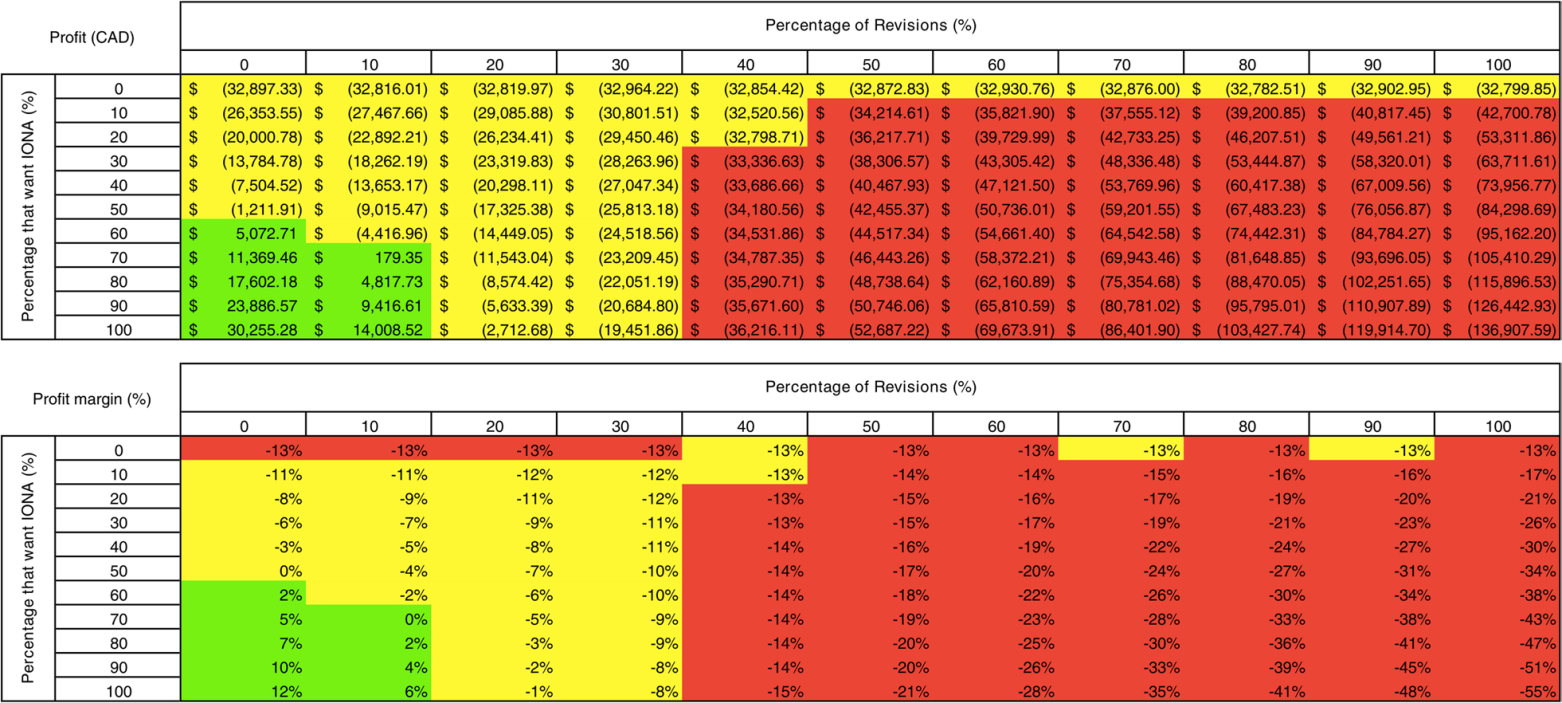
Financial sensitivity analysis. Effect of (1) percentage of patients who opt to receive IONA (rows) and (2) percentage of IONA revisions (columns) on the proposed state profit (CAD) and profit margin (%). For the profit table, green indicates profit > 0, yellow indicates 0 > profit > current state profit, and red indicates profit < current state profit. For the profit margin table, green indicates profit margin > 0, yellow indicates 0 > profit margin > current state profit margin, and red indicates profit margin < current state profit margin

Financial sensitivity analysis revealed that the proposed state profit was higher than the current state profit if as few as 10% of patients selected IONA, with the maximum revision rate needing to remain below 40% to achieve improved profits (Fig. 3). A positive profit was reached when the percentage of patients who selected IONA reached 60% and a maximum revision rate of 10%. The maximum profit achievable in the proposed state, with 100% of patients selecting IONA and 0% revision rates, was $ 30,255.28. Similarly, the profit margin of the proposed state was higher than the current state with as little as 10% selecting IONA, with the maximum revision rate remaining below 40%. Again, a positive profit margin was reached when at least 60% of patients selected IONA, with a maximum revision rate of 10%. The maximum profit margin in the proposed state was 12%.

**Fig. 4:**
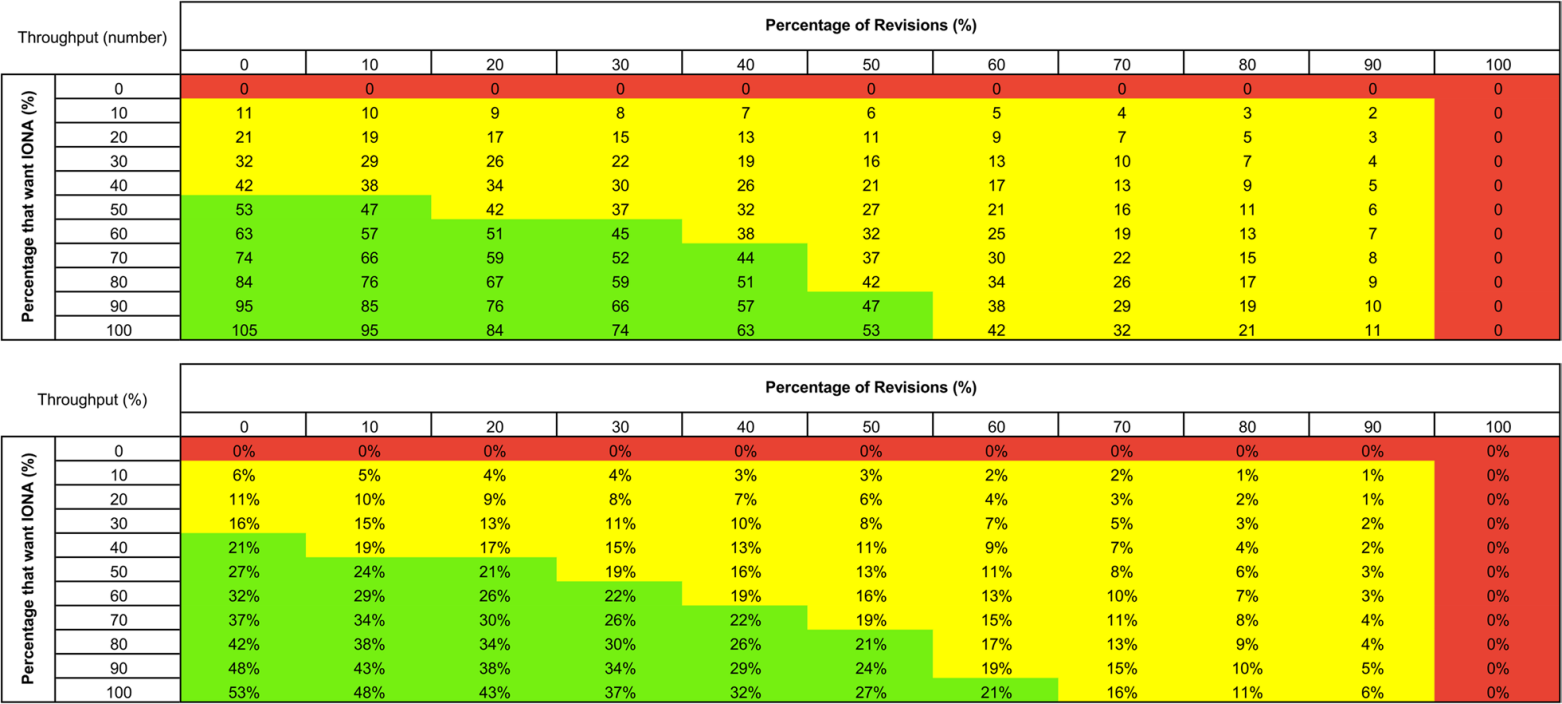
Throughput sensitivity analysis. Effect of (1) percentage of patients who opt to receive IONA (rows) and (2) percentage of IONA revisions (columns) on the throughput (numbers) and throughput (%). For both the numbers and percentages tables, green indicates throughput > heuristics model throughput, yellow indicates heuristics model throughout > throughput > 0, and red indicates throughput < 0 for numbers and percentages, respectively

Throughput sensitivity analysis revealed that the maximum increase in throughput possible with the proposed state was 105 patients, representing a 53% improvement in throughput. As expected, any number of patients selecting IONA will result in an improvement in throughput (Fig. 4).

## Discussion

The most important finding from this study is that IONA is a cost-effective alternative to traditional surgical arthroscopy for medial meniscus meniscectomy. In addition to decreasing direct hospital costs and reducing surgical wait times, it is also estimated that the reduced waitlists will relieve indirect costs, including disability payments and time off work, and improve patient satisfaction. Given the perilous state of orthopedic surgery costs and waiting times, OR diversion via IONA is an idea worth further exploring.

Utility values of partial arthroscopic meniscectomy attempt to estimate the health-related quality of life for cost-effectiveness analysis purposes. These values include the gamut of possible outcomes following a procedure using the Standard Gamble (1 meaning perfect health, 0 meaning death) and are typically utilized to compare the cost-utility of procedures for a given condition. The gain in quality-adjusted life years (QALY) from partial meniscectomy is reported as a range of 8.09–17, leading to an incremental cost-utility ratio gain of $853-$1793/QALY in the proposed state model [36–38]. Arthroscopic knee meniscectomy has historically been deemed not cost-effective in most cases, but the savings from IONA may begin to shift this perspective.

Sensitivity analysis revealed that only a small portion of patients (10%) needed to select IONA with a maximum revision rate of 40% would yield a profit greater than the current state. If the number of patients who select IONA increased to at least 60% while the revision rate remained below 10%, a positive profit would be achieved. This has profound implications for hospital administrators and decision-makers as it demonstrates the poor finances of the current state while highlighting an underutilized pathway to increase profitability. It not only provides a solution to increased profits but provides a care pathway whereby the throughput of patients can be increased without compromising care, something desperately needed in modern constrained healthcare systems. Furthermore, the use of ten thousand Monte Carlo Simulations allows one to consider the variance in patient volumes over time, solidifying the results of the financial analysis. This is important as hospital administrators need to understand the full financial impact of implementing such a care pathway diversion, something which this study supports.

This study used secondary data to estimate that 20.3% of patients booked for IONA for expected medial meniscus resection based on MRI would have repairable tears. Subtle factors on MRI such as the location of tear, type of tear, and level of certainty add further variables that can influence the decision to offer IONA. Clinical expertise suggests that this number may be far lower in actual practice, as predicted repairability is a composite judgment involving patient age, injury characteristics, and other sociodemographic factors [39]. Thus, the reported savings (reduction in expenses) of $ 14,502.55 may overestimate the proportion of patients requiring subsequent operative meniscal repair and thereby underestimate the expected savings.

The concept of OR diversion has seen widespread financial success in other surgical fields, but is frequently met with concerns of patient safety, particularly in the circumstance of eventual hospital transfer. Provincial QBP data suggest that the rate of serious complications is exceedingly low in uncomplicated outpatient arthroscopy for meniscus procedures [23]. Once again, surgeon discretion should be applied when offering IONA to patients with a catastrophic knee injury, psychosocial discomfort, prior surgical/anesthetic, complications, or serious medical comorbidities [40, 41]. Unforeseen concerns with OR diversion, such as sterility, pain control, and decreased airway support, may be encountered [40]. The efficacy, complication rates, and patient-reported satisfaction from IONA are yet to be reported in any primary research studies [42].

Importantly, IONA can also be used as a diagnostic procedure. It is shown to be a cost-effective alternative to MRI with similar diagnostic accuracy. The role of IONA as a joint diagnostic-therapeutic tool could positively impact MRI waiting times and MRI/MRA costs, and further reduce indirect costs to society. Given the well-established benefit of early meniscus treatment, accelerating both diagnosis and therapy has the potential to reduce costs and improve patient quality of life [15, 17, 40, 43–46].

The primary limitation of this work is its reliance on secondary data to provide estimates for the prevalence of isolated medial meniscal tears, predicted repairability, and success of treatment. Surgical decision-making regarding repairability involves a host of patient- and injury-related factors, including age, level of activity, symptoms, type and location of tear, chronicity, and intraoperative assessment of healing. When pooled analysis was not possible, the authors utilized data that overestimated repairability rates (e.g., studies in the traumatic meniscal tear population), thereby potentially understating the financial benefits of IONA. Second, the analysis did not consider patients who receive IONA indicating repair but chose to have an IONA meniscectomy instead. This would require further assumptions from the literature that are not available but may strengthen the case for IONA as a cost-effective approach. Third, the revision rate for incomplete meniscectomies using IONA, i.e., where an unstable flap remained with persistent symptoms post IONA meniscectomy, was not accounted for. This is limited by paucity in the literature, which supports further studies investigating the efficacy IONA. Fourth, there are no primary studies reporting the safety and efficacy outcomes of IONA medial meniscectomy. It is possible that this procedure could differ in outcomes from OR medial meniscectomy in terms of visualization, access, or completeness of resection, and that patient preference, procedure length, and other qualitative factors could influence the cost-effectiveness and feasibility of implementation. This limitation fuels further work: that a primary clinical trial comparing IONA and OR arthroscopy is necessary to verify the theoretical cost–benefit identified in this study. A further limitation is that these models do not account for the possible lost clinic time by adding IONA procedures to clinic. As there is not yet an established approach on how to incorporate IONA into clinic—for example, having a dedicated IONA clinic versus integrating into a standard clinic day—this was left for future analysis. In addition, these models are particular to a community hospital in a Canadian healthcare system and so the conclusions are not necessarily directly applicable to other hospitals and healthcare systems. However, the modeling approach is generalizable and thus may be applied to other contexts while making minor adjustments such as costs and remuneration. Finally, the false negative rates of meniscus tear on MRI were difficult to quantify due to the lack of repairability values. Therefore, it was assumed that this cost would be equivalent in the present and proposed states, and it was not included in any of the DuPont tree calculations.

## Conclusion

IONA is a cost-effective alternative to traditional OR arthroscopy in patients undergoing partial medial meniscectomy in a Canadian community hospital setting. Reduced OR wait times are likely to have myriad downstream benefits to society, including decreased time off work, reduced disability payments, and greater patient satisfaction. The next steps are to qualitatively assess patients’ perceptions and attitudes of IONA as an alternative to traditional OR arthroscopy, and to carry out clinical trials to determine the efficacy, patient-reported outcome metrics, and complications of IONA.

## Data Availability

All data gathered in this study are available in the full text.

## Abbreviations

IONA: In-office needle arthroscopy
MCS: Monte Carlo simulation
OR: Operating room
PMM: Partial medial meniscectomy
QBP: Quality-based procedure
SD: Standard deviation
